# Visceral Fat, Metabolic Health, and Lifestyle Factors in Obstructive Bronchial Diseases: Insights from Bioelectrical Impedance Analysis

**DOI:** 10.3390/nu17061024

**Published:** 2025-03-14

**Authors:** Ștefana-Oana Popescu, Andreea Mihai, Adina Turcu-Știolică, Carmen Elena Lupu, Diana-Maria Cismaru, Victor Ionel Grecu, Alexandru Scafa-Udriște, Răzvan Ene, Magdalena Mititelu

**Affiliations:** 1 Department of Biochemistry, Faculty of Medicine, University of Medicine and Pharmacy of Craiova, 200349 Craiova, Romania; oana.popescu@umfcv.ro; 2 Municipal Hospital Orșova, 225200 Orșova, Romania; 3 Pharmaceutical Management and Marketing, Faculty of Pharmacy, University of Medicine and Pharmacy of Craiova, 200349 Craiova, Romania; adina.turcu@umfcv.ro; 4 Department of Mathematics and Informatics, Faculty of Pharmacy, “Ovidius” University of Constanta, 900001 Constanta, Romania; 5 National School of Political Studies and Public Administration, College of Communication and Public Relations, 012104 Bucharest, Romania; diana.cismaru@comunicare.ro; 6 Victor Babeș Clinical Hospital for Infectious Diseases and Pneumophthisiology, 200515 Craiova, Romania; grecuvictorionel@yahoo.com; 7 Department of Cardio-Thoracic Pathology, Faculty of Medicine, “Carol Davila” University of Medicine and Pharmacy, 050474 Bucharest, Romania; alexandru.scafa@umfcd.ro; 8 Clinical Department No. 14, Faculty of Medicine, “Carol Davila” University of Medicine and Pharmacy, 050474 Bucharest, Romania; razvan.ene@umfcd.ro; 9 Department of Clinical Laboratory and Food Safety, Faculty of Pharmacy, “Carol Davila” University of Medicine and Pharmacy, 020956 Bucharest, Romania; magdalena.mititelu@umfcd.ro

**Keywords:** metabolic syndrome, obstructive bronchial diseases, chronic obstructive pulmonary disease, asthma, dietary habits, physical activity, body mass index, lipid profile, respiratory health

## Abstract

**Background/Objectives**: This study examines the relationship between visceral fat (VF), metabolic health, and dietary patterns in patients with obstructive bronchial diseases (OBDs) using bioelectrical impedance analysis (BIA). **Methods**: A total of 75 patients diagnosed with OBD, including chronic obstructive pulmonary disease (COPD) and/or asthma, were assessed for VF levels via BIA. Dietary habits were evaluated using a structured questionnaire to explore their correlation with VF accumulation. **Results**: The study cohort comprised predominantly male participants (66.7%), with the majority aged between 61 and 70 years (46.7%). Significant gender differences in VF distribution were observed, with 60% of females maintaining normal VF levels (1–9) compared to only 28% of males, while 38% of males exhibited very high VF levels (15–30; *p* = 0.003). Body mass index (BMI) showed a strong correlation with VF (*p* < 0.0001), as overweight and obese individuals predominantly displayed elevated VF levels (≥10). Moreover, metabolic syndrome (MS) was present in 66.7% of participants, with these individuals exhibiting significantly higher VF levels compared to those without MS (*p* = 0.001). Dietary analysis revealed that frequent consumption of fast food (r = 0.717, *p* < 0.001), carbonated drinks (r = 0.366, *p* = 0.001), and refined carbohydrates (r = 0.438, *p* < 0.001) was significantly associated with increased VF accumulation. Conversely, higher intake of water (r = −0.551, *p* < 0.001), fruits (r = −0.581, *p* < 0.001), and vegetables (r = −0.482, *p* < 0.001) correlated with lower VF levels. Lack of physical activity was also strongly linked to VF accumulation (r = 0.481, *p* < 0.001), further reinforcing the role of lifestyle factors in metabolic health. **Conclusions**: The findings underscore the significant impact of dietary habits and physical activity on VF accumulation in OBD patients. BMI and MS emerged as critical predictors of VF, while unhealthy dietary patterns and sedentary lifestyles further exacerbated VF deposition. Elevated VF levels were linked to adverse lipid profiles, reinforcing the need for dietary and lifestyle modifications in managing metabolic health among OBD patients. Although no direct association was identified between VF and forced expiratory volume in one second (FEV1), the results highlight the necessity of integrated nutritional and metabolic interventions in the management of chronic respiratory diseases.

## 1. Introduction

The global obesity epidemic has escalated to unprecedented levels, affecting over one billion individuals worldwide—approximately one in eight people—as of 2022 [1]. This rising trend is expected to continue, with projections indicating that by 2030, nearly 57.8% of the global adult population will be classified as overweight or obese [2,3]. These alarming figures underscore the pressing need for effective, evidence-based strategies to combat obesity, address its underlying causes, and mitigate its increasing impact on healthcare systems worldwide.

In addition to its role as an energy reservoir, adipose tissue functions as a dynamic endocrine organ, releasing a variety of bioactive molecules known as adipokines. These adipokines are essential regulators of appetite, metabolism, systemic inflammation, and immune responses [4,5]. However, the physiological impact of adipose tissue largely de-pends on its distribution within the body. Classified into visceral and subcutaneous fat, these two compartments exhibit distinct metabolic properties, each contributing differently to the health risks associated with obesity [6,7].

Visceral obesity, defined by excessive fat accumulation around internal organs, is strongly associated with metabolic disorders such as hypertension, cardiovascular dis-ease, and type 2 diabetes. It also increases the risk of malignancies, including prostate, colon, and breast cancers and cardiovascular disease [8,9,10,11]. Understanding the distinct roles of different adipose tissue types is important for developing targeted interventions against obesity-related complications.

Metabolic dysfunction-associated steatotic liver disease (MASLD) is closely linked to metabolic syndrome (MS), with key contributing factors including type 2 diabetes, dyslipidemia, and excess weight. Among these, abdominal obesity plays a particularly significant role, impacting respiratory health through mechanical compression of the diaphragm and systemic inflammation.

Patients with MASLD also exhibit increased susceptibility to infections due to immune dysfunction, gut dysbiosis, and metabolic disturbances such as hyperglycemia, which impairs neutrophil function. As MASLD progresses to severe stages like cirrhosis, the risk of infections escalates due to systemic immunodeficiency, known as cirrhosis-associated immune dysfunction syndrome (CAIDS). A recent meta-analysis encompassing data from 26.6 million participants revealed that individuals with MASLD have a significantly elevated risk of developing severe bacterial infections, with this risk escalating in tandem with the degree of liver fibrosis [12]. Although the precise mechanisms underlying this increased susceptibility remain incompletely understood, several contributing factors have been identified. These include impaired hepatic clearance of microbes, reduced levels of vitamin D, dysfunction of immune cells (e.g., natural killer cells and Kupffer cells), and insulin resistance-related impairments in neutrophil function. Additional factors such as small intestinal bacterial overgrowth and increased intestinal permeability further exacerbate infection risk [13,14,15].

In this context, MASLD patients with comorbid conditions such as chronic obstructive pulmonary disease (COPD) and/or asthma face an even greater infection risk. These respiratory disorders are characterized by impaired immune responses and heightened susceptibility to respiratory infections. The coexistence of pulmonary comorbidities amplifies the overall risk profile, significantly increasing the likelihood of severe infections, hospitalizations, and mortality. This underscores the urgent need for vigilant management and close monitoring of this vulnerable population to mitigate the compounded risks of severe infections and associated complications [16,17].

Dysfunctional adipose tissue associated with abdominal obesity significantly alters the secretion profile of adipokines, promoting a state of chronic, low-grade systemic inflammation. This persistent inflammatory milieu disrupts pulmonary immune responses and exacerbates airway hyper-reactivity, thereby worsening respiratory conditions [18]. Addressing abdominal visceral obesity effectively requires accurate assessment methods for the visceral fat (VF) area. While waist circumference (WC) is a convenient and widely used metric for estimating fat accumulation, it lacks precision in distinguishing between visceral and subcutaneous fat, limiting its utility for accurately isolating VF [19,20].

Advanced imaging modalities such as computed tomography (CT) and magnetic resonance imaging (MRI) offer precise quantification of VF and remain the gold standards for VF assessment. However, their routine clinical use is constrained by several challenges, including high costs, the need for specialized expertise to interpret results, and, in the case of CT, exposure to ionizing radiation. These limitations make such techniques im-practical for widespread use in many healthcare settings [8,21].

Bioelectrical impedance analysis (BIA) has become a practical, non-invasive, and cost-effective tool for assessing body composition, including fat mass, lean body mass, and hydration levels. BIA measures the resistance of body tissues to electrical currents—fat-free mass, rich in water and electrolytes, conducts electricity efficiently, while fat mass, an insulator, exhibits higher resistance. By incorporating parameters such as height, weight, age, and gender, BIA provides reliable estimates of VF distribution, making it a valuable tool in clinical, fitness, and research settings.

Body composition consists of fat mass and fat-free mass, each playing an essential role in metabolic health. While essential fats support physiological functions, excessive stored fat increases the risk of cardiovascular disease (CVD) and diabetes. Conversely, fat-free mass, comprising muscles, bones, water, and organs, is vital for metabolic rate, mobility, and overall physical strength. Imbalances in fat distribution can have significant health consequences, with VF accumulation strongly linked to MS, hypertension, and insulin resistance [22,23,24,25].

BIA facilitates early detection of metabolic risk factors by estimating VF levels and assessing the fat-to-lean mass ratio, providing insights into metabolic dysfunction. In conditions such as diabetes, excess fat deposition in non-adipose tissues exacerbates metabolic disruption. Additionally, BIA detects changes in skeletal muscle mass, an important factor since sarcopenia is a strong predictor of adverse outcomes in CVD, including heart failure and stroke. Moreover, by evaluating extracellular and intracellular water levels, BIA aids in identifying fluid imbalances, a key complication in heart failure linked to poor prognosis [26,27,28,29,30].

Given its affordability, accuracy, and ease of application, BIA represents an accessible alternative to advanced imaging techniques for routine clinical use, particularly in evaluating metabolic health and cardiovascular disease risk. Personalized interventions based on BIA assessments—ranging from dietary modifications and exercise regimens to medical therapies—can optimize patient outcomes by reducing fat mass, preserving lean body mass, and improving metabolic function.

COPD significantly impacts metabolic health, increasing the risk of CVD, type 2 diabetes, and obesity through interconnected mechanisms [31,32,33,34]:

Systemic inflammation: COPD-induced chronic inflammation extends beyond the lungs, with elevated levels of pro-inflammatory cytokines (e.g., IL-6, TNF-α) impairing insulin sensitivity and promoting VF accumulation, both of which are hallmarks of metabolic dysfunction.

Sarcopenia and muscle wasting: Common in advanced COPD, sarcopenia reduces physical activity, exacerbates insulin resistance, and heightens the risk of metabolic dis-eases. Muscle loss also contributes to the development of MS and increases cardiovascular risk.

Fat distribution and hormonal dysregulation: COPD patients may exhibit both cachexia and obesity, particularly central obesity, which is closely linked to systemic inflammation and MS. Hormonal imbalances, such as dysregulated leptin and adiponectin levels, further exacerbate metabolic dysfunction and insulin resistance.

BIA plays an important role in monitoring these changes by assessing the fat-to-muscle ratio, tracking sarcopenia progression, and identifying VF accumulation. These insights can guide tailored interventions to mitigate metabolic dysfunction and improve outcomes in COPD patients.

Nutritional status and physical activity exert a profound influence on VF accumulation, particularly in individuals with obstructive bronchial diseases (OBDs), such as COPD and asthma. Suboptimal dietary habits, including excessive caloric intake, high consumption of processed and ultra-processed foods rich in trans fats and refined sugars, and an inadequate intake of essential micronutrients, contribute to metabolic dysregulation and visceral adiposity. These dietary patterns promote chronic low-grade inflammation, insulin resistance, and dyslipidemia, all of which are strongly linked to increased VF deposition. Additionally, a sedentary lifestyle, characterized by prolonged inactivity and reduced engagement in structured physical exercise, further exacerbates this accumulation by impairing lipid oxidation, reducing muscle mass, and diminishing overall metabolic efficiency.

Conversely, a well-balanced diet—emphasizing whole foods, lean proteins, unsaturated fats, and complex carbohydrates—plays a crucial role in modulating VF levels and improving metabolic health. Specific dietary components, such as omega-3 fatty acids, polyphenols, and dietary fiber, have been shown to reduce systemic inflammation and enhance lipid metabolism, thereby mitigating VF accumulation. Likewise, regular physical activity, including both aerobic and resistance training, promotes energy expenditure, enhances mitochondrial function, and facilitates the mobilization of stored VF. Exercise-induced adaptations, such as improved insulin sensitivity and increased anti-inflammatory cytokine production, further contribute to the reduction of VF and the prevention of metabolic complications.

Given the well-established relationship between excessive VF and MS, cardiovascular risk, and systemic inflammation, optimizing both nutrition and physical activity is essential for the comprehensive management of OBD patients. Lifestyle interventions aimed at improving dietary quality and increasing physical activity levels should be integrated into pulmonary rehabilitation programs to not only reduce VF accumulation but also enhance respiratory function, minimize disease exacerbations, and improve overall patient outcomes.

This study aims to identify key contributors to the increased risk of MS in this population and emphasizes the critical role of VF assessment in clinical practice. VF accumulation is influenced by dietary habits, such as the intake of processed foods, refined carbohydrates, and sugar-sweetened beverages, as well as physical activity levels. Balanced nutrition and regular exercise are essential for maintaining metabolic health and reducing VF deposition. This research seeks to bridge the knowledge gap regarding the relationship between VF, MS, and pulmonary function, particularly forced expiratory volume in one second (FEV1), and underscores the need for integrating nutritional and metabolic assessments into pulmonology care. The study highlights the potential of BIA as a non-invasive, cost-effective tool to evaluate VF levels, providing a more comprehensive framework for managing OBD patients and emphasizing the need for personalized, multidisciplinary interventions to improve their health and quality of life.

Based on these observations, this study aims to investigate the relationship between VF accumulation and various clinical and demographic factors in patients with chronic obstructive pulmonary diseases (COPDs) and/or asthma. By analyzing these associations, we seek to identify key contributors to the increased risk of MS in this patient population. Furthermore, this research underscores the critical role of VF assessment in routine clinical practice, emphasizing its potential to enhance the management of OBD patients by guiding personalized therapeutic and lifestyle interventions. Dietary habits and physical activity levels are key determinants of VF accumulation, with excessive intake of processed foods, refined carbohydrates, and sugar-sweetened beverages contributing to increased VF deposition, while balanced nutrition and regular physical exercise support metabolic homeostasis. Understanding the interplay between VF, MS, and pulmonary function is essential for optimizing clinical management strategies, as excessive VF may exacerbate systemic inflammation and worsen respiratory outcomes.

## 2. Materials and Methods

This study was designed as a cross-sectional, non-interventional investigation involving patients diagnosed with chronic obstructive pulmonary diseases. Participants were recruited from the pulmonology departments of two hospitals in the Oltenia Region of Romania—Leamna Clinical Pneumophthisiology Hospital and The Clinical Hospital of Infectious Diseases and Pneumophthisiology “Victor Babeș” Craiova—during the period between March and July 2023.

Participation in the study was entirely voluntary, with all subjects providing informed consent following a comprehensive explanation of the study’s objectives and procedures. The study adhered strictly to the ethical principles outlined in the most recent version of the Declaration of Helsinki. Ethical approval was obtained from the respective healthcare institutions prior to patient enrollment (Leamna Clinical Pneumophthisiology Hospital and The Clinical Hospital of Infectious Diseases and Pneumophthisiology “Victor Babeș” Craiova) and by the Ethics Committee of the University of Medicine and Pharmacy of Craiova. All participants’ data were handled in compliance with applicable data protection regulations, and the study was conducted without any discrimination based on social status, political affiliation, or religious beliefs.

This study included adult patients (≥18 years) with a confirmed diagnosis of COPD and/or asthma, established through spirometry and clinical evaluation according to current official guidelines. Only individuals in a stable clinical condition, without acute exacerbations of their pulmonary disease in the past four weeks, were eligible for inclusion. To ensure consistency and accuracy in data collection, participants were recruited during periods of clinical stability, allowing for an objective evaluation of clinical, paraclinical, and respiratory function parameters while minimizing potential confounders such as infectious exacerbations or acute systemic conditions.

Exclusion criteria encompassed patients presenting with acute respiratory infections, recent exacerbations of their pulmonary disease, or any other acute illnesses at the time of recruitment. Furthermore, individuals with severe systemic diseases—such as advanced cardiovascular conditions, end-stage renal disease, or active malignancies—that could significantly alter body composition or pulmonary function were excluded. Additional exclusion factors included contraindications for spirometry, conditions leading to abdominal distension that might interfere with anthropometric measurements, and any physical or cognitive impairments that would hinder adherence to the study protocol.

All participants provided written informed consent after receiving a comprehensive explanation of the study’s objectives, methodology, and potential risks.

The primary aim of this research was to evaluate the utility of BIA in correlating with lipid profile parameters and pulmonary function metrics. Additionally, the study sought to investigate BIA’s predictive potential for identifying MS in patients with chronic pulmonary conditions treated in a pulmonology department.

### 2.1. Study Design

This study aimed to investigate the relationship between VF and various factors in patients with OBD. It explored the association between VF and MS, assessing whether MS criteria and serum triglycerides (TGL) levels correlate with elevated VF. The study also examined the impact of VF on pulmonary function (FEV1) and evaluated WC and BMI as potential markers for VF. Additionally, it considered gender differences in VF levels and the influence of nutrition and physical activity on VF accumulation, seeking to understand how these factors contribute to metabolic health in OBD patients.

The presence of MS was determined using the revised guidelines of the National Cholesterol Education Program—Adult Treatment Panel III (NCEP-ATP III). All assessments were carried out according to these specified guidelines. According to this definition, MS is identified by meeting at least three of the following five criteria [35,36,37,38]:

MS 1. Abdominal obesity: WC of ≥102 cm for men; ≥88 cm for women.
MS 2. High TGL: TGL level of ≥150 mg/dL or under treatment for elevated TGL.
MS 3. Low high-density lipoprotein (HDL) cholesterol: <40 mg/dL for men or treatment for low HDL cholesterol; <50 mg/dL for women or treatment for low HDL cholesterol.
MS 4. Elevated blood pressure (BP): systolic BP (SBP) ≥ 130 mmHg and/or diastolic BP (DBP) ≥ 85 mmHg, or on antihypertensive medication.
MS 5. High blood glucose level: fasting glucose of ≥100 mg/dL or on antidiabetic medication.

### 2.2. Extensive Evaluation: Anamnesis, Anthropometric Measurements, Bioimpedance-Based Body Composition Analysis, Biological Sample Collection, Blood Pressure Monitoring, and Pulmonary Function Testing

Anthropometric measurements began with height determination, using a stadiometer with patients standing upright, without footwear. With patients in light clothing, an individualized body composition analysis was conducted using a bioimpedance-based medical scale, the OMRON Body Composition Monitor BF511 HBF-511T-E/HBF-511B-E (OMRON HEALTHCARE Company; Kyoto, Japan). This included measuring body weight, BMI, VF percentage, and resting metabolism (RM), with the device requiring the input of the participant’s age, gender, and height for accurate determination. Subsequently, WC was assessed at the level of the umbilicus, with the patient in a standing position and at the point of full expiration [39]. The Omron BF511 body composition monitor utilizes 8 electrodes for measurements: 4 grip electrodes for the hands and 4 foot electrodes for the feet. This configuration ensures full-body assessment, reducing the impact of water distribution changes throughout the day on the results.

The biological sample collection involved obtaining approximately 10 mL of venous blood after an overnight fast and before administering any chronic medication. These samples were analyzed using an automatic device—BioSystems A15 (BioSystems Company, Barcelona, Spain)—to determine fasting blood glucose, serum TGL, and HDL cholesterol levels.

BP measurements, including systolic and diastolic values, were obtained using a certified automated electronic monitor (OMRON X3 Comfort HEM-7155-E; Omron Company; Kyoto, Japan) following standard protocol.

The pulmonary function assessment of the study participants was systematically conducted through spirometry using the Spirolab IV (Medical International Research Company; Rome, Italy). In addition to ensuring patient safety and the procedure’s accuracy, we strictly adhered to guidelines to obtain reliable results by confirming the absence of smoking and using medications that could have influenced the test in the hours preceding the spirometry.

All participants completed a standardized semiquantitative questionnaire designed to systematically assess lifestyle and dietary habits, with a particular focus on eating patterns and unhealthy behaviors. The survey included alcohol consumption and physical activity levels. A validated tool was used to evaluate behavioral risk factors, particularly those linked to the excessive consumption of ultra-processed foods and sugar-sweetened beverages, known to have detrimental health effects [26,36].

The questionnaire quantified the frequency and intake of both nutrient-dense foods with protective health benefits—such as fiber, probiotics, antioxidants, vitamins, minerals, essential amino acids, and fatty acids—and unhealthy dietary choices, including ultra-processed foods high in saturated fats and refined carbohydrates (e.g., junk food and sweetened beverages).

The survey was conducted through an online platform (Google Forms) to ensure standardized data collection. To enhance response accuracy and reliability, a trained physician was responsible for administering the questionnaire. The physician provided verbal explanations of each question, clarified response options, and recorded answers using an assigned identification number for each participant, ensuring data integrity.

### 2.3. Data Analysis

We performed descriptive statistics; continuous variables were presented as mean ± standard deviation (DS), median (interquartile range, IQR), and range (minimum and maximum), whereas discrete variables were presented as frequencies and percentages. We tested for the normality of the continuous variables with the Shapiro–Wilk test (the test rejects the hypothesis of normality when the *p*-value is less than or equal to 0.05), and violin plots were used to visually demonstrate their normality. The Wilcoxon–Mann–Whitney test was used to compare two groups non-parametrically when normally distributed data were not met. The heatmap of correlations was used to evaluate the associations between continuous variables. A *p*-value of less than 0.05 was considered statistically significant.

To facilitate data processing, patients were categorized into the following age groups: (1) under 50 years, (2) 51 to 60 years, (3) 61 to 70 years, (4) 71 to 80 years, and (5) over 80 years. This classification aids in simplifying the analysis and identifying age-related trends within the study.

Additionally, based on the cut-off values for VF provided by the scale manufacturer, patients were categorized using distinct VF levels (normal, high, very high). This approach further refines the analysis by aligning it with standardized measures and thresholds for VF assessment. According to the manufacturer of the device used in the study, Omron Healthcare, the unit for VF measured by BIA is referred to as a VF rating or index. VF is expressed as a score (e.g., 1–9 indicating low, 10–14 indicating high, and 15–30 indicating very high), which provides a classification of the degree of VF accumulation and serves as a risk stratification tool.

Statistical power analysis determined that a sample size of 64 participants would be necessary to detect a moderate effect size (Cohen’s d = 0.5) with 80% power at a 5% significance level [40]. Given our sample size of 75, this study has adequate statistical power to detect meaningful associations.

We performed Principal Component Analysis (PCA) to explore the relationships between VF levels and dietary habits such as the consumption of vegetable products, fruits, fish, water, fast food, sweets or pastries, bread, sweetened and carbonated drinks, and alcohol, as well as physical activity.

The quantification of the frequency of consumption of certain food products and lifestyle habits was carried out using the following codifications: Fast food (FF) consumption was quantified as 1—representing very rare or no consumption, 2—consumption two to three times a month, 3—consumption once a week, 4—consumption two to three times a week, and 5—daily consumption. Vegetable (Veg) and fruit (Fr) consumption was measured as 1—indicating more than three portions of 100 g per day, 2—three portions per day, 3—two portions per day, 4—one portion per day, and 5—very rare or no consumption. Sweetened and carbonated drinks (SD) were quantified as 1—very rare or no consumption, 2—two to three times a month, 3—once a week, 4—two to three times a week, 5—daily consumption of one portion (330 mL per portion), and 6—daily consumption of more than one portion. Alcohol (Alc) consumption was measured as 1—very rare or no consumption, 2—two to three times a month, 3—once a week, 4—two to three times a week, 5—daily consumption of one portion, and 6—daily consumption of more than one portion. Fish and seafood (Fish) consumption was quantified as 1—daily consumption, 2—two to three times a week, 3—once a week, 4—two to three times a month, and 5—very rare or no consumption. The consumption of sweets and pastries (Sw/part) was measured as 1—very rare or no consumption, 2—two to three times a month, 3—once a week, 4—two to three times a week, and 5—daily consumption. Bread consumption (Br) was quantified as 1—very rare or no consumption, 2—one to four slices per day, 3—five to seven slices per day, 4—eight to twelve slices per day, and 5—more than twelve slices per day. Water (Wat) consumption was measured as 1—more than three liters per day, 2—three liters daily, 3—two liters daily, 4—one liter daily, and 5—less than one liter daily. Physical activity (Ex) was quantified as 1—daily exercise of at least one hour, 2—daily exercise of less than one hour, 3—two to three times a week, 4—very rare exercise, and 5—no exercise.

In the Supplementary Materials, the components of the diet (macro- and micronutrients), the impact on human health, and food sources are presented in Table S1. This table helps to understand the assessment of the impact of nutrition on the health of the patients in the study, but also to establish recommendations regarding the balancing of nutrition for curative purposes.

## 3. Results

Of the 75 patients diagnosed with obstructive bronchial pathology, 66.7% (n = 50) were male. The largest proportion of patients (46.7%) fell within the 61–70 age group, while 12.0% were under the age of 50. Older age groups were less represented, with 16% of patients aged 71–80 years and 8.0% over the age of 80. The majority of the cohort (69.3%) were retired, and residency was nearly equally divided between urban (50.7%) and rural (49.3%) areas. Pulmonary diagnoses included 58.7% with COPD and 41.3% with asthma. Regarding BMI, 33.3% of patients were classified within the normal weight range, 32.0% were overweight, and 30.6% were obese. Notably, 66.7% of the patients met the minimum criteria for MS, highlighting a significant prevalence of associated metabolic abnormalities within this cohort.

### 3.1. The Distribution of Visceral Fat Levels Related to Socio-Demographic Factors

The distribution of VF levels across age groups reveals an age-related trend, although the *p*-value of 0.135 indicates that these differences do not reach statistical significance, as shown in Table 1. In contrast, significant gender-based differences in VF levels were observed. Among females (n = 25), the majority (60%) had normal VF levels, 36% exhibited high VF, and only 4% had very high VF levels. Conversely, among males (n = 50), only 28% presented with normal VF, 34% had high VF levels, and a substantial proportion (38%) had very high VF. These findings suggest that males are more likely to exhibit elevated VF levels compared to females, with the *p*-value of 0.003 confirming that the gender-based differences in VF distribution are statistically significant.

An examination of pulmonary disease types revealed that patients with COPD (58.7% of the sample) exhibited the following VF distribution: 40.9% with normal VF, 34.1% with high VF, and 25% with very high VF. Asthma patients (41.3% of the sample) had a similar distribution, with 35.5% presenting normal VF, and equal proportions (35.5% each) with high VF and 29% with very high VF. The *p*-value of 0.878 suggests no significant difference in VF distribution between COPD and asthma patients, indicating that the type of pulmonary disease does not significantly affect VF levels.

A statistically significant correlation between BMI and VF levels was identified, with a *p*-value of less than 0.0001. This indicates that BMI is closely associated with VF accumulation. Underweight and normal-weight individuals predominantly exhibited normal VF levels, with 84% of normal-weight individuals maintaining healthy fat profiles. However, the overweight category showed a marked shift, with 79.2% presenting with high VF, a trend that continued in the obese category. This clear stratification underscores the strong association between increasing BMI and escalating VF accumulation, with implications for metabolic health and disease risk.

The relationship between MS status and VF accumulation demonstrated a significant association, with a *p*-value of 0.001. Among participants without MS (33.3% of the sample), 68% exhibited a healthier distribution of VF (VF ≤ 9), suggesting a protective effect of the absence of MS against excessive VF accumulation and its associated health risks. In contrast, individuals diagnosed with MS (66.7% of the sample) exhibited significantly higher VF levels. Specifically, only 24% of this group maintained normal VF levels, while 40% were classified as having high VF (≥10), and 36% presented with very high VF levels (≥15). This marked disparity emphasizes the detrimental impact of MS on VF deposition, highlighting that the presence of metabolic risk factors notably increases the likelihood of excessive VF accumulation (Table 1).

The latest guidelines for classifying COPD are outlined in the “Global Strategy for Prevention, Diagnosis, and Management of COPD: 2025 Report” published by the Global Initiative for Chronic Obstructive Lung Disease (GOLD). This report is updated annually to incorporate the latest scientific evidence and advancements, providing a comprehensive framework for assessing disease severity, guiding therapeutic strategies, and optimizing patient outcomes. The GOLD classification system categorizes COPD based on the severity of airflow limitation as measured by forced expiratory volume in one second (FEV1) [41]: 1. early stages of COPD (GOLD 1–2); 2. moderate stages of COPD (GOLD 3); 3. advanced stages of COPD (GOLD 4).

FEV1-based classification remains a cornerstone in the clinical evaluation of COPD, offering a standardized approach to guide individualized management and therapeutic interventions. Stratifying VF according to COPD stage underscores its dynamic relationship with disease progression and highlights the importance of tailored interventions to address the evolving metabolic and inflammatory risks associated with COPD [42].

The distribution of VF is different across patients with and without MS for patients with GOLD 2 and GOLD 4. The distribution of VF is the same across patients with and without MS for patients with GOLD 3 (Table 2).

The incidence of MS by GOLD stages in the analyzed group of patients is presented in Table 3. No significant correlation was observed between GOLD stage and FAT among patients without MS (Spearman’s rho = −0.04, *p* > 0.05). However, a moderate correlation was identified among patients with MS (Spearman’s rho = −0.42, *p* < 0.05).

### 3.2. Visceral Fat Distribution Analysis

The descriptive statistics for VF reveal that in the sample consisting of 75 participants, the median VF value was 11.0, and the mean was 11.58. The standard deviation of 6.441 suggests moderate variability. Skewness was slightly positive at 0.660, showing a right-skewed distribution, while kurtosis was near zero (0.056), indicating a distribution similar to normal. The Shapiro–Wilk test (W = 0.956, *p* = 0.010) showed that the VF distribution deviates significantly from normality, with values ranging from 1 to 29.

#### 3.2.1. Metabolic Syndrome Status in Relation to Visceral Fat Level

**Figure 1:**
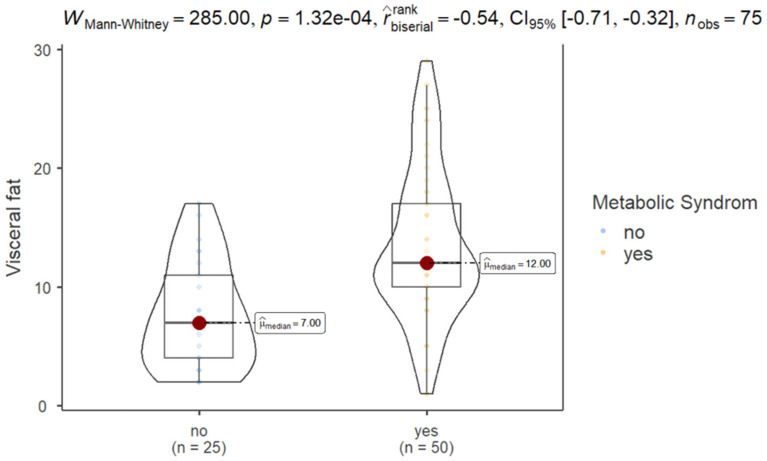
Comparison of VF among patients with and without MS.

The results of Mann–Whitney test showed that patients with MS exhibited significantly higher VF (mean ± SD, 13.5 ± 6.41) compared to those without MS (7.68 ± 4.49), as shown in Figure 1.

#### 3.2.2. Metabolic Syndrome Criteria in Relation to Visceral Fat Level

Group-specific statistics show that participants without MS had a mean VF of 7.680 (SD = 4.488), while those with MS had a higher mean VF of 13.540 (SD = 6.412). The coefficient of variation was slightly lower for the MS group (0.474) compared to the non-MS group (0.584), indicating relatively lower variability in VF levels among those with MS.

The distribution of VF levels across different MS criteria highlights significant gender-based differences. For patients classified as MS 0, indicating no MS criteria, all individuals (100%) had normal VF levels. As the severity of MS increased, there was a marked shift towards higher VF levels (Table 4).

In the MS 1 group, which consisted entirely of men, 60% had normal VF, while 40% had high levels, as shown in Table 4.

For the MS 2 category, most patients (64.7%, including 66.7% of females and 63.6% of males) had normal VF. Conversely, 23.5% of the MS 2 group (33.3% of females and 18.2% of males) had high VF levels. Additionally, 11.8% of male patients in MS 2 exhibited very high VF levels.

In the MS 3 group, 42.9% of patients had normal VF levels, with a notable gender difference: 83.3% of females and only 12.5% of males. Conversely, 35.7% of patients had VF, affecting 16.7% of females and 50% of males. Notably, the very high VF category was exclusively observed in males, with 37.5% of them affected.

Among individuals with MS 4, only 16.7% had normal VF levels, with a gender distribution of 33.3% females and 11.1% males. High VF was observed in 50% of patients, with a notable gender disparity: 66.7% of males and 44.4% of females. The significant proportion of males (44.4%) with high VF in this category highlights an increased risk associated with advanced MS stages. This underscores a gender-dependent difference in VF distribution, with males showing a higher prevalence of high VF levels as MS severity increases.

Finally, in the MS 5 group, only 44.4% of females (representing 16.7% of the total MS 5 population) and none of the males had normal VF levels. High VF was present in 44.4% of females and 33.3% of males, accounting for 37.5% of the MS 5 cohort. Notably, 11.1% of females and most males (66.7%) exhibited very high VF levels. These data underscore a pronounced gender disparity, with males showing a significantly higher prevalence of very high VF in the most severe stage of MS.

The observed *p*-value of 0.022 indicates that the associations between MS severity and VF levels are statistically significant. This suggests a meaningful relationship between the progression of MS and VF distribution. As the severity of MS increases, there is a notable shift in VF levels, with higher stages of MS correlating with greater amounts of VF.

**Figure 2:**
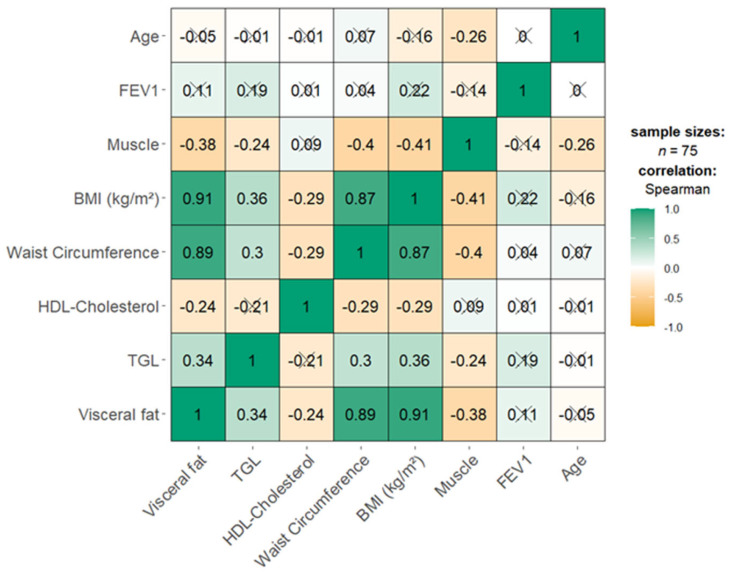
The heatmap shows the correlations between visceral fat, triglycerides (TGL), HDL cholesterol, waist circumference, BMI, muscle, FEV1, and age. Bright green indicates strong positive correlations (Spearman’s rho = 1), while bright orange signifies strong negative correlations (Spearman’s rho = −1), X = non-significant at *p* < 0.05.

We identified a significant negative correlation between VF and HDL cholesterol (Spearman’s rho = −0.239, *p* = 0.039), indicating that higher VF is associated with lower HDL cholesterol levels, as shown in Figure 2.

A significant positive correlation was observed between VF and TGL (Spearman’s rho = 0.343, *p* = 0.003), suggesting that increased VF level is linked to higher TGL levels, as shown in Figure 1.

Furthermore, strong and significant positive correlations were observed between VF and both WC (Spearman’s rho = 0.891, *p* < 0.001) and BMI (Spearman’s rho = 0.907, *p* < 0.001), as illustrated in Figure 2.

#### 3.2.3. The Influence of Gender on Visceral Fat

**Figure 3:**
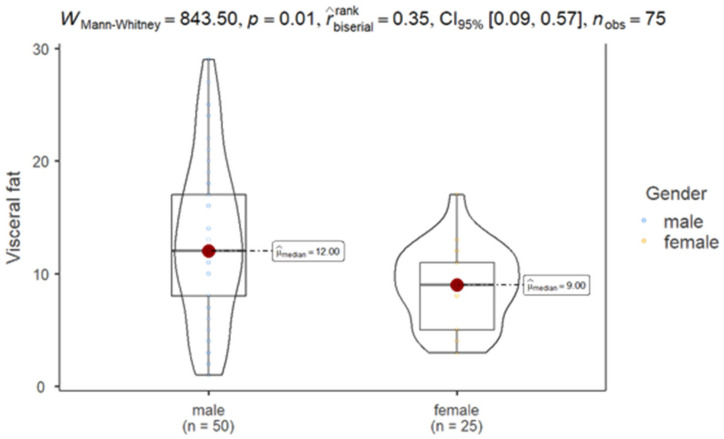
Comparison of VF by gender.

The results of the Mann–Whitney test showed significantly lower VF levels in females compared to males (*p* = 0.01), validating the idea that gender influences VF distribution (Figure 3).

### 3.3. Comparison of Triglycerides, Waist Circumference, Body Mass Index, Forced Expiratory Volume in One Second, Number of Criteria for Metabolic Syndrome, and HDL Cholesterol in Determining Visceral Fat Levels in Patients with Obstructive Bronchial Diseases

**Figure 4:**
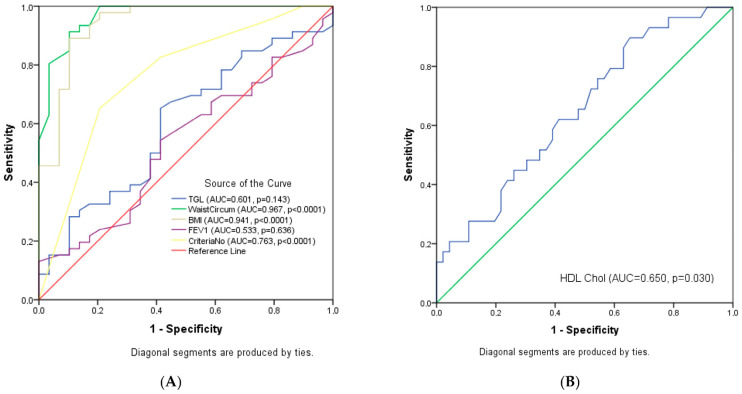
(**A**) ROC curve analysis for comparison of TGL, WC, BMI, FEV1, and the number of criteria for MS in determining VF levels in patients with OBD. (**B**) ROC curve analysis for HDL cholesterol in determining VF levels in patients with OBD.

Younden’s Index was negative for FEV1 and number of criteria, indicating a flaw in the testing process or misinterpreting results. The ROC curves showed that WC [AUC = 0.967, *p* < 0.0001] and BMI [AUC = 0.941, *p* < 0.0001] have the greatest capacity to identify patients with obstructive pulmonary diseases and high VF levels, as shown in Figure 4.

Table 5 presents the diagnostic accuracy of various test parameters for identifying high VF levels among patients with OBD. WC and BMI stand out with the highest diagnostic performance, having AUC values of 0.967 and 0.941, respectively, both highly significant (*p* < 0.0001). The optimal WC cut-off is ≥96.0, achieving high sensitivity (93.5%) but low specificity (17.2%), with a Younden’s Index of 0.107. Similarly, the optimal BMI cut-off of ≥24.9 yields a sensitivity of 91.3% and specificity of 17.2%, with a Younden’s Index of 0.095, suggesting moderate diagnostic utility.

TGL showed limited diagnostic relevance, with an AUC of 0.601 and a non-significant *p*-value (0.143), indicating a weak association with high VF levels. HDL cholesterol demonstrated modest diagnostic value, with an AUC of 0.650 (*p* = 0.030) and an optimal cut-off of ≤45.65, resulting in a Younden’s Index of 0.123. Both FEV1 and the number of MS criteria showed poor diagnostic performance, with FEV1 displaying an AUC of 0.533 (*p* = 0.636) and a negative Younden’s Index (−0.043), suggesting no diagnostic value. The number of criteria showed a slightly better AUC of 0.763 (*p* < 0.0001) but still had a negative Younden’s Index (−0.141), highlighting its limited diagnostic ability.

Overall, WC and BMI are the most effective indicators for identifying high VF levels in this patient group.

### 3.4. Correlation Between the Criteria Number for Metabolic Syndrome and Visceral Fat, Triglycerides, HDL Cholesterol, Forced Expiratory Volume in One Second, Body Mass Index, and Waist Circumference

A strong positive correlation was observed between VF and the criteria number for MS (Spearman’s rho = 0.539, *p* < 0.001), indicating that increased VF was associated with a higher likelihood of MS criteria (Table 6). Similarly, TGL showed a strong positive correlation (rho = 0.552, *p* < 0.001), suggesting that elevated TGL levels are linked to an increased risk of MS. In contrast, HDL cholesterol demonstrates a moderate negative correlation (rho = −0.478, *p* < 0.001), indicating that higher HDL levels are associated with a lower criteria number for MS. Notably, BMI also shows a strong positive correlation (rho = 0.557, *p* < 0.001), indicating that higher BMI is associated with a greater likelihood of meeting MS criteria. Lastly, WC exhibits a robust positive correlation (rho = 0.632, *p* < 0.001), further underscoring the relationship between increased waist size and an elevated criteria number for MS. In summary, higher VF, TGL, BMI, and WC are significantly associated with a greater likelihood of meeting MS criteria, while HDL cholesterol is inversely related.

### 3.5. Correlation Between Nutrition, Physical Activity, and VF

**Figure 5:**
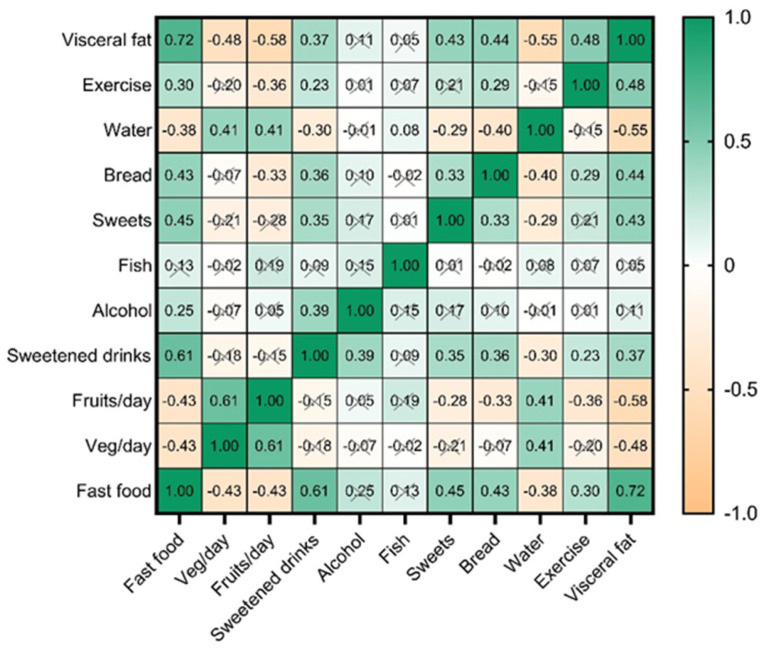
The heatmap showing the correlation between visceral fat, dietary intake (fast food, sweets, bread, sweetened drinks, fruits, vegetables, fish, water consumption), and physical activity. Bright green indicates strong positive correlations (Spearman’s rho = 1), while bright orange signifies strong negative correlations (Spearman’s rho = −1). X = non-significant at *p* < 0.05.

Analysis of the correlations between VF and coded dietary habits (Figure 5) revealed significant associations between frequent consumption of fast food, sweets, pastries, carbonated drinks, and high levels of VF (VF_3), highlighting the negative impact of these foods on metabolism. A negative correlation was also observed between adequate water intake, frequent consumption of fruits, vegetables, and fish and lower levels of VF (VF_1 and VF_2).

Significant positive correlations with VF:

Fast food (FF): Fast food consumption is strongly associated with higher levels of VF (r = 0.717, *p* < 0.001), highlighting the negative impact of processed and calorie-dense foods on VF accumulation.

Carbohydrate drinks (SD): There is a moderate and significant correlation between carbonated beverage consumption and VF (r = 0.366, *p* = 0.001), reflecting the role of sugar in increasing VF.

Bread (Br): Bread consumption is positively associated with VF (r = 0.438, *p* < 0.001), suggesting that a high intake of simple carbohydrates may contribute to VF accumulation.

Lack of physical exercise (Ex): Inactive individuals have higher levels of VF (r = 0.481, *p* < 0.001), highlighting the importance of physical activity in preventing VF accumulation.

Significant negative correlations with VF:

Water (Wat): Water consumption is negatively associated with VF (r = −0.551, *p* < 0.001), suggesting that adequate hydration may play a protective role against VF accumulation.

Fruit (Fr): There is a strong negative correlation between fruit consumption and VF (r = −0.581, *p* < 0.001), reflecting the benefits of a diet rich in fiber and vitamins.

Vegetables (Veg): Vegetable consumption is also negatively associated with VF (r = −0.482, *p* < 0.001), highlighting the importance of a high intake of fiber and essential nutrients in the daily diet.

Correlations between dietary variables:

Fast food and carbonated drinks: There is a strong correlation between fast food and carbonated drink consumption (r = 0.609, *p* < 0.001), indicating an unhealthy dietary pattern, characterized by high calorie and sugar intake.

Fruits and vegetables: Fruit consumption is positively associated with vegetable consumption (r = 0.607, *p* < 0.001), reflecting balanced eating behavior and awareness of the importance of a varied diet.

Water and vegetables: People who consume more water tend to include more vegetables in their diet (r = 0.411, *p* < 0.001), suggesting a healthy lifestyle and awareness of the importance of hydration.

Fruit and fish: There is a moderate correlation between fruit and fish consumption (r = 0.255, *p* = 0.027), indicating a diversified and healthy eating behavior, which may contribute to a balanced intake of essential nutrients, such as omega-3 fatty acids.

Negative correlations between dietary variables:

Water and sweets: Water consumption is negatively associated with sweet consumption (r = −0.290, *p* = 0.012), suggesting that well-hydrated individuals tend to consume fewer foods rich in sugar.

Water and bread: Water consumption is also negatively associated with bread consumption (r = −0.400, *p* < 0.001), reflecting a tendency to avoid foods rich in simple carbohydrates.

Fast food and lack of physical exercise: There is a moderate correlation between fast food consumption and lack of physical exercise (r = 0.301, *p* = 0.009), suggesting that people with a sedentary lifestyle tend to consume more fast food.

Carbonated drinks and bread: Carbonated drink consumption is positively associated with bread consumption (r = 0.364, *p* = 0.001), indicating a dietary pattern rich in simple carbohydrates and empty calories.

**Figure 6:**
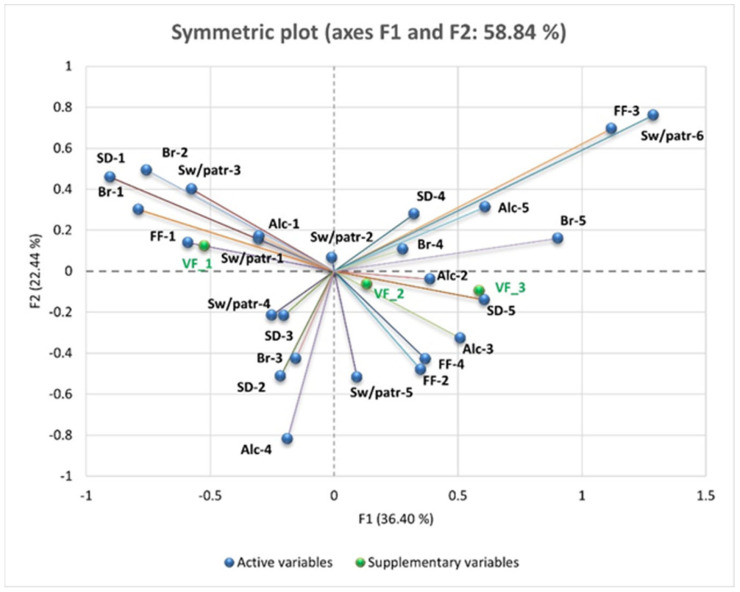
PCA of VF levels (VF_1, VF_2, and VF_3), dietary intake (fast food, sweets or pastries, bread, sweetened and carbonated drinks, and alcohol). Fast food (FF-4 and FF-5), sweets and pastries (Sw/patr-4 and Sw/patr-5), and sweetened drinks (SD-5 and SD-6) are linked with F2, while most variables are associated with F1. Legend: FF = fast food; Sw/patr = sweets and pastries; SD = sweetened/carbonated drinks; Alc = alcohol; Br = bread.

**Figure 7:**
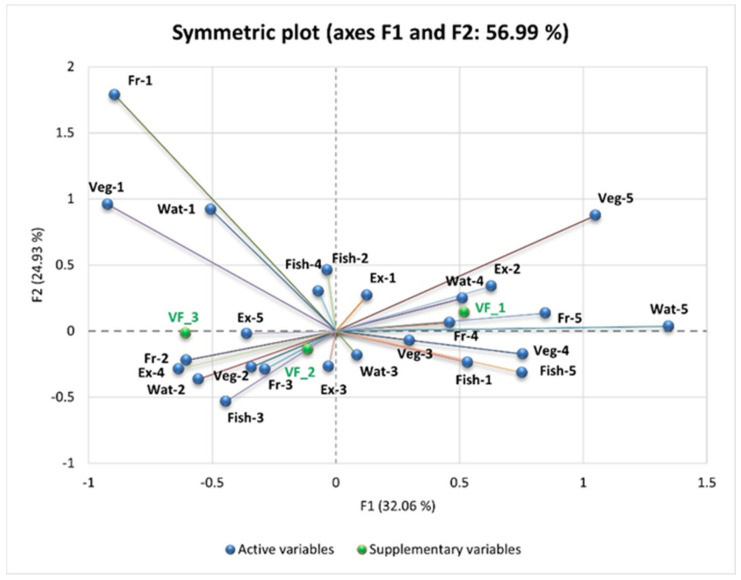
PCA of VF levels (VF_1, VF_2, and VF_3), healthy diet (vegetable products, fruits, water consumption, and fish), and physical activity. Fruits (Fr-1 and Fr-2), vegetables (Veg-1, Veg-2, and Veg-4), and water consumption (Wat-1) are linked with F2, while most variables are associated with F1. Legend: veg = vegetable products; fr = fruits; wat = water; fish = fish and seafood; ex = physical activity.

To complement the correlation analysis, PCA was performed to explore multidimensional relationships between VF levels and coded dietary habits. As shown in the PCA (Figure 6 and Figure 7), individuals with higher levels of VF (VF_3) tend to have higher scores for frequent consumption of fast food, sweets, pastries, and sweetened beverages—indicating an unbalanced diet rich in fats and sugars. These individuals also exhibit higher scores for low vegetable and fruit consumption and insufficient water intake—reflecting an inadequate diet. In contrast, individuals with lower levels of VF (VF_1 and VF_2) show higher scores for frequent consumption of vegetables, fruits, and fish or seafood; reduced consumption of fast food, sweets, and carbonated drinks; optimal hydration; and regular physical activity. This highlights the association between a healthy lifestyle and reduced VF levels, while unhealthy dietary habits contribute to its accumulation.

PCA highlights the relationships between the consumption of “unhealthy” foods and VF (VF_1—Normal VF level, VF_2—High VF level, and VF_3—Very high VF level), explaining 56.99% of the total variation in the data (F1: 32.06% and F2: 24.93%).

VF_3 is strongly positively correlated with the frequent consumption of fast food (FF-5, “Daily”), sweets and pastries (Sw/patr-5, “Daily”), and alcoholic beverages (Alc-5, “Daily one serving”).

VF_1 is negatively correlated with these unhealthy eating habits and is positively associated with regular physical exercise (SD-1, “Yes, daily at least one hour”) and reduced consumption of sweets (Sw/patr-1, “Very rarely or not at all”).

VF_2 is negatively correlated with frequent consumption of bread (Br-5, “More than 12 slices/day”) and carbonated or sweetened beverages (SD-6, “Daily more than one serving”). Reducing the consumption of refined carbohydrates and added sugars is associated with lower levels of VF.

VF_3 is positively correlated with low fruit and vegetable intake. People who consume very little or no fruit (Fr-5) and very little or no vegetables (Veg-5) tend to have higher levels of visceral fat.

Water intake also plays a key role. Low water intake (Wat-5, less than 1 L/day) is associated with higher VF_3 values, while intake of more than 3 L/day (Wat-1) is negatively correlated with VF, highlighting the benefits of adequate hydration on metabolism and fat storage.

VF_1 is positively correlated with daily exercise (Ex-1, “Yes, daily for at least one hour”) and moderate water intake (Wat-4, 2 L/day). This indicates that regular physical activity and adequate hydration are associated with low VF. VF_2 is negatively correlated with a low intake of fish (Fish-5, “Very rarely or not at all”) and fruit (Fr-3, “Two servings/day”).

## 4. Discussion

This study sought to evaluate VF levels using BIA in patients diagnosed with OBD. The analysis indicated that VF levels were similar between urban and rural residents, suggesting that residential environment may not play a substantial role in VF distribution.

An examination of educational levels in relation to VF distribution suggested minor variations, though no clear pattern emerged. Notably, participants with an intermediate educational attainment (ISCED 4, comprising 25.3% of the cohort) exhibited a greater tendency for favorable VF outcomes, with 42.1% maintaining normal VF levels, suggesting potential benefits linked to intermediate education (Table 1).

Regarding the relationship between pulmonary disease type and VF categories, patients with COPD, which comprised 58.7% of the sample, displayed a VF distribution of 40.9% with normal VF, 34.1% with high VF, and 25% with very high VF. Similarly, asthma patients (41.3% of the sample) showed a comparable VF distribution: 35.5% with normal VF, 35.5% with high VF, and 29% with very high VF levels.

VF distribution appeared similar between COPD and asthma patients, suggesting that pulmonary disease type may not have a notable impact on VF levels in this cohort.

A clear relationship was observed between BMI and VF levels, with higher BMI corresponding to increased VF accumulation.

This suggests that variations in BMI are closely correlated with VF accumulation. Underweight and normal-weight individuals primarily exhibited normal VF levels, with 84% of normal-weight individuals maintaining healthy fat profiles. In contrast, the overweight category demonstrated a substantial shift, with 79.2% presenting high VF levels, a trend that continued in the obese category. This clear stratification of risk emphasizes the relationship between increased BMI and elevated VF accumulation, highlighting the implications for metabolic health and disease risk.

An analysis of occupational status suggested no notable differences in VF levels among the groups. The findings indicated a clear association between MS and VF accumulation, suggesting a potential link between metabolic risk factors and increased VF levels.

Among participants without MS, who accounted for 33.3% of the sample, 68% exhibited normal VF levels, suggesting a protective effect of the absence of MS against excessive VF accumulation and its associated health risks. Conversely, individuals with MS (66.7% of the sample) displayed significantly higher VF levels, with only 24% maintaining normal VF levels. Specifically, 40% were categorized as having high VF, and 36% presented with very high VF levels. This marked disparity underscores the adverse impact of MS on VF deposition, indicating that the presence of metabolic risk factors notably increases the likelihood of excessive VF accumulation.

We observed that 60% of females had normal VF levels, while only 28% of males exhibited similar levels. Additionally, 38% of males were classified as having very high VF levels, highlighting a significant gender disparity (*p* = 0.003) and suggesting that males with OBD are more prone to elevated VF levels compared to females. A complementary retrospective cross-sectional study conducted at Yuxi People’s Hospital, involving 1882 patients, also identified pronounced gender differences in VF distribution. Notably, males, despite being generally younger, demonstrated significantly higher VF areas than females [43]. The authors attributed this to hormonal influences; specifically, testosterone promotes abdominal fat accumulation in males, while estrogens in females lead to fat storage in the hips and thighs [44,45].

These findings align closely with our results, suggesting that males are at a greater risk for VF accumulation, which may contribute to adverse health outcomes.

This study establishes a significant association between BMI and VF levels, indicating a close relationship between variations in BMI and VF accumulation in this patient population. Furthermore, the findings suggest a strong link between the presence of MS and VF accumulation, emphasizing the increased risk of excessive VF in individuals with metabolic risk factors.

Notably, VF levels tended to be higher in participants with more severe MS, suggesting a relationship between the extent of metabolic dysfunction and VF accumulation.

Similarly, a Taiwanese cross-sectional study demonstrated that elevated VF level is a strong independent predictor of MS in both men and women, with odds ratios of 1.33 and 1.28, respectively [46].

Moreover, this finding aligns with another study involving 1451 Japanese residents aged 30 to 69 examining the utility of BIA in measuring VF level showed that participants diagnosed with MS exhibited significantly higher mean VF values—12.1 in men and 13.3 in women—compared to their non-MS counterparts, who had mean values of 9.4 and 8.7, respectively [47].

In a study conducted at Walailak University Hospital involving ninety participants, equally divided between men and women aged 18 to 60 years, all with a BMI of 25 kg/m^2^ or higher, researchers identified significant correlations between VF levels and metabolic indicators. Notably, positive associations were found with serum TGL (r = 0.287, *p* = 0.006) and fasting blood glucose (r = 0.210, *p* = 0.047), while a negative correlation with HDL cholesterol was observed (r = −0.322, *p* = 0.002). These results align with our findings, which also demonstrated similar relationships between VF, TGL, and HDL cholesterol among patients with obstructive bronchial conditions, emphasizing the detrimental impact of VF on metabolic health across diverse cohorts [48].

In our study, we observed no direct relationship between VF and FEV1, a key lung function indicator. Conversely, a separate community-based study in Shanghai involving a cohort of 1786 individuals aged 40 and older who underwent lung function testing found that a higher visceral adiposity index (VAI) was associated with a decrease in FEV1% predicted, particularly in women [49].

These discrepancies may arise from the VAI’s calculation in this study, which utilized a formula integrating WC, TGL, and HDL cholesterol, more likely serving as a metric of metabolic risk. Furthermore, the comparative study excluded individuals with chronic lung conditions, which might have influenced their findings.

In contrast, a study conducted in Xinjiang involving 2367 participants found that while the mean VF determined by a body component analyzer was significantly higher in males compared to females, the relationship between VAI and lung function varied by sex. Specifically, in males with VF below a certain threshold (14–15), higher VAI levels were positively associated with improved FEV1 and FVC. However, above this threshold, an increase in VF correlated with a decline in these lung function measures. This inverted U-shaped relationship suggests that VF may have differing impacts on lung function based on both sex and the degree of fat accumulation, further complicating the understanding of its role in respiratory health [50].

The findings suggest that VF levels do not appear to be directly linked to COPD severity as classified by the GOLD system.

However, the previous literature and studies indicate a more complex relationship between VF and COPD, particularly in its advanced stages.

It has been observed that the accumulation of VF in COPD is often associated with more severe dyspnea, as indicated by the MMRC (Modified Medical Research Council) scale, which might further limit physical activity and lead to a cycle of fat accumulation and muscle wasting [51].

A study of 294 COPD patients, in which VF was assessed in the android region using DEXA, found that both male and female patients with more severe airflow limitation (GOLD 3/4) had significantly lower VF values [52].

This aligns with findings from other research that conducted body composition assessments using CT scans, revealing a more nuanced relationship between fat distribution and COPD severity. Specifically, patients with progressed COPD (GOLD 2–3) exhibited lower VF levels, while increased bronchial wall thickening was associated with greater VF accumulation. This suggests that the distribution of fat, rather than its total volume, may have distinct impacts on lung function, particularly in the context of bronchial wall thickening [53]. This reduction in VF in advanced COPD stages could be attributed to cachexia, a condition marked by weight loss and muscle depletion, which is common in the late stages of COPD. Despite these findings, when stratified by BMI, VF values were similar among patients with moderate to very severe COPD [52]. Further research provides additional insight into the relationship between COPD severity and body composition. The findings of a recent study, which assessed baseline chest CT scans from 1031 male participants in a lung cancer screening program, indicated that COPD was associated with reduced levels of subcutaneous fat, VF, and skeletal muscle. As the GOLD stages progressed, there was an increase in subcutaneous fat and VF, while skeletal muscle decreased. Importantly, higher emphysema severity correlated with lower fat and muscle values, whereas bronchial wall thickening was linked to increased fat accumulation. These associations remained significant even after adjusting for age, smoking history, and pack-years, emphasizing the role of COPD and emphysema severity in altering body composition [54].

Moreover, another research focusing on older COPD patients with abdominal obesity highlighted that increased visceral adipose tissue was associated with relatively preserved lean mass, muscle function, and physical activity levels. However, these individuals also exhibited higher levels of systemic inflammation and metabolic comorbidities, which may explain the increase in VF despite a decline in muscle mass. This suggests that increased VF in advanced COPD may not only be a result of physical inactivity but also a reflection of broader metabolic dysfunction [55].

Understanding these dynamics is essential for tailoring future patient management strategies in respiratory health.

The analysis of dietary habits and VF accumulation in patients with OBD reveals significant associations between poor dietary choices and excessive VF levels.

The findings highlight a strong link between fast food consumption and increased VF levels, emphasizing the potential metabolic risks of processed, high-calorie diets. Similarly, frequent intake of carbonated beverages and bread appears to contribute to VF accumulation, suggesting that excessive sugar and refined carbohydrates may play a role in VF deposition.

Physical inactivity is another key factor influencing VF levels. The positive correlation between lack of exercise and VF reinforces the well-established link between sedentary lifestyles and increased adiposity. Individuals with low physical activity levels tend to consume more fast food, further compounding their risk of metabolic dysfunction.

Conversely, a diet rich in fruits, vegetables, and adequate water intake appears to exert a protective effect against excessive VF accumulation. The results suggest that higher fruit and vegetable consumption may support metabolic regulation and help reduce VF levels, likely due to their fiber and micronutrient content. Additionally, adequate hydration appears to play a role in lipid metabolism, as greater water intake is associated with lower VF accumulation.

The interrelationship between dietary habits further emphasizes distinct nutritional patterns. The tendency for fast food and carbonated drink consumption to occur together suggests a clustering of unhealthy dietary behaviors, while the positive association between fruit and vegetable intake reflects a more balanced and health-conscious approach. Additionally, the inverse relationship between water consumption and both sweets and bread intake indicates that individuals with better hydration habits may be less inclined toward sugary and refined carbohydrate-rich foods.

These findings underscore the critical role of diet and physical activity in managing visceral adiposity and its associated metabolic risks. Given the elevated prevalence of MS among patients with OBD, integrating tailored nutritional interventions and structured physical activity programs into disease management strategies could significantly mitigate disease progression and improve overall patient outcomes. Future research should explore the longitudinal impact of dietary modifications and exercise regimens on VF reduction to establish evidence-based lifestyle recommendations for this vulnerable patient population.

The PCA results, illustrated in Figure 6 and Figure 7, reveal distinct dietary patterns associated with varying VF levels. Individuals classified under the highest VF category (VF_3) exhibited a strong positive association with frequent consumption of fast food, sweets, pastries, and sugar-sweetened beverages, indicating a dietary profile rich in fats and refined sugars. These individuals also had lower vegetable and fruit intake and insufficient water consumption, reinforcing the connection between poor dietary habits and increased VF accumulation.

Conversely, individuals in the lower VF categories (VF_1 and VF_2) demonstrated healthier dietary patterns. Higher consumption of vegetables, fruits, and fish, coupled with reduced intake of fast food, sweets, and carbonated beverages, was associated with lower VF levels. These individuals also maintained optimal hydration and engaged in regular physical activity, underscoring the protective effect of a balanced lifestyle against excessive VF deposition.

The PCA model accounted for 56.99% of the total variance in the dataset, with the first principal component (F1) explaining 32.06% and the second principal component (F2) capturing 24.93%. Notably, VF_3 was strongly correlated with daily fast food consumption (FF-5), sweets and pastries (Sw/patr-5), and alcoholic beverages (Alc-5), reinforcing the detrimental impact of high-caloric, processed foods on visceral adiposity. In contrast, VF_1 was negatively associated with these unhealthy eating habits and positively correlated with daily physical activity (Ex-1) and minimal sweets consumption (Sw/patr-1), suggesting that exercise and dietary moderation contribute to lower VF levels.

Additionally, VF_2 was negatively correlated with excessive bread intake (Br-5) and frequent consumption of carbonated or sweetened beverages (SD-6). This finding highlights the adverse effect of refined carbohydrates and added sugars on VF accumulation. Similarly, individuals with higher VF_3 values exhibited lower fruit (Fr-5) and vegetable (Veg-5) consumption, emphasizing the metabolic benefits of a plant-based diet.

Hydration also emerged as a significant factor in VF regulation. Low water intake (Wat-5, less than 1 L/day) was positively correlated with VF_3, whereas the consumption of more than 3 L/day (Wat-1) was negatively associated with VF, supporting the role of adequate hydration in metabolic health. Furthermore, regular physical activity (Ex-1) and moderate water intake (Wat-4, 2 L/day) were positively linked to VF_1, reinforcing the synergistic effects of exercise and hydration in maintaining optimal body composition.

The observed trends suggest that targeted lifestyle modifications—such as reducing processed food intake, increasing fruit and vegetable consumption, maintaining proper hydration, and engaging in consistent physical activity—may be effective strategies in mitigating VF accumulation and its associated metabolic risks.

Based on the findings of this study, a multidisciplinary approach incorporating tailored dietary and physical activity interventions is recommended to improve the quality of life in patients with OBD. Specifically:

Physical activity promotion: Given the significant correlation between sedentary behavior and increased VF, structured exercise programs should be integrated into pulmonary rehabilitation strategies. A combination of aerobic exercises (e.g., walking, cycling, or swimming) and resistance training can help improve metabolic health, reduce VF accumulation, and enhance lung function. Moderate-intensity physical activity for at least 150 min per week should be recommended, with adaptations based on individual respiratory capacity.

Comprehensive lifestyle education: Educational programs focusing on dietary awareness, meal planning, and physical activity should be included in routine patient management. Behavioral counseling and patient-centered coaching can reinforce adherence to healthy habits, ultimately reducing VF-related complications and improving long-term respiratory and metabolic health.

Clinical monitoring and personalized care: Regular assessment of VF levels using BIA alongside metabolic and respiratory evaluations should be incorporated into routine care. Personalized interventions based on dietary intake, physical activity levels, and metabolic risk factors can enhance treatment efficacy and contribute to a more holistic approach to OBD management.

By integrating these evidence-based dietary and exercise recommendations into standard pulmonary care, clinicians can help OBD patients achieve better metabolic health, reduce systemic inflammation, and improve overall quality of life.

### Limitations

This study has several limitations that warrant consideration. The relatively small sample size of 75 participants may compromise the statistical power and generalizability of the findings. The cross-sectional design restricts causal interpretations, as it captures VF levels at a single time point. Moreover, the reliance on BIA for VF assessment may lack the precision of imaging modalities such as MRI or CT scans.

Another possible limitation of the study is determined by the degree of subjectivity in the responses collected from the questionnaire assessing the dietary habits and lifestyle of the patients enrolled in the study.

## 5. Conclusions

This study demonstrates that VF plays an essential role in metabolic disturbances and systemic inflammation in patients with OBD, particularly COPD and/or asthma. Given its strong association with MS criteria and unfavorable lipid profiles, VF should be considered a key factor in disease progression and overall health outcomes in this patient population. The findings also support the use of BIA as a practical, non-invasive tool for VF assessment in clinical settings. Moreover, the influence of dietary habits on VF accumulation underscores the need for targeted lifestyle interventions to mitigate metabolic risk factors in OBD patients. Although no direct link was observed between VF levels and lung function (FEV1), the complex relationship between adiposity and respiratory health warrants further investigation. Future research should explore the mechanistic pathways connecting VF accumulation with pulmonary dysfunction, systemic inflammation, and oxidative stress to improve disease management strategies.

## Figures and Tables

**Table 1:** Clinical and demographic data classified by visceral fat levels.

Characteristics	Categories	Total		Normal Visceral Fat Level		High Visceral Fat Level		Very High Visceral Fat Level		*p*-Value
		N = 75	%	N = 29	%	N = 26	%	N = 20	%	
Age	<50	9	12.0	6	66.7	1	11.1	2	22.2	0.135
	51–60	13	17.3	4	30.8	4	30.8	5	38.5	
	61–70	35	46.7	13	37.1	10	28.6	12	34.3	
	71–80	12	16.0	4	33.3	7	58.3	1	8.3	
	>80	6	8.0	2	33.3	4	66.7	0	0	
Gender	F	25	33.3	15	60	9	36	1	4	0.003
	M	50	66.7	14	28	17	34	19	38	
Residence	Urban	38	50.7	16	42.1	12	31.6	10	26.3	0.798
	Rural	37	49.3	13	35.1	14	37.8	10	27	
Educational level	ISCED 0–2	41	54.7	14	34.1	16	39	11	26.8	0.714
	ISCED 3	12	16	6	50	3	25	3	25	
	ISCED 4	19	25.3	8	42.1	7	36.8	4	21.1	
	ISCED 5–6	2	2.7	1	50	0	0	1	50	
	ISCED 7–8	1	1.3	0	0	0	0	1	100	
Pulmonary disease	COPD	44	58.7	18	40.9	15	34.1	11	25	0.878
	Asthma	31	41.3	11	35.5	11	35.5	9	29	
BMI	Underweight	3	4.0	3	100	0	0	0	0	<0.0001
	Normal weight	25	33.3	21	84	4	16	0	0	
	Overweight	24	32	3	12.5	19	79.2	2	8.3	
	Obesity I	16	21.3	2	12.5	3	18.8	11	68.8	
	Obesity II	3	4.0	0	0	0	0	3	100	
	Obesity III	4	5.3	0	0	0	0	4	100	
Occupational status	Employed	11	14.7	5	45.5	1	9.1	5	45.5	0.259
	Unemployed	12	16.0	6	50	4	33.3	2	16.7	
	Retired	52	69.3	18	34.6	21	40.4	13	25	
MS status	No	25	33.3	17	68	6	24	2	8	0.001
	Yes	50	66.7	12	24	20	40	18	36	

**Table 2:** The distribution of VF by GOLD stages in the analyzed group of patients.

GOLD	Patients with SM N = 29	Patients Without SM N = 15	*p*-Value
2 (n = 17)	16.46 ± 7.22	6.75 ± 2.5	0.006
3 (n = 14)	12.7 ± 8.19	8.0 ± 5.35	0.374
4 (n = 13)	12.33 ± 3.61	5.86 ± 3.98	0.014

No significant correlation was found between VF and GOLD stage (rho = −0.22, *p*-value > 0.05).

**Table 3:** The incidence of MS by GOLD stages.

GOLD	Patients with SM N = 29	Patients Without SM N = 15	Total N = 44
2	13 (44.8%)	4 (26.7%)	17 (38.6%)
3	10 (34.5%)	4 (26.7%)	14 (31.8%)
4	6 (20.7%)	7 (46.7%)	13 (29.5%)

**Table 4:** VF levels in relation to MS criteria and gender distribution.

	MS0		MS1		MS2		MS3		MS4		MS5		*p*-Value
	F (N = 1)	M (N = 2)	F (N = 0)	M (N = 5)	F (N = 6)	M (N = 11)	F (N = 6)	M (N = 8)	F (N = 3)	M (N = 9)	F (N = 9)	M (N = 15)	
	%	%	%	%	%	%	%	%	%	%	%	%	
Normal VF	100	100	0	60	66.7	63.6	83.3	12.5	33.3	11.1	44.4	0	0.022
High VF				40	33.3	18.2	16.7	50	66.7	44.4	44.4	33.3	
Very high VF						18.2		37.5		44.4	11.1	66.6	

Visceral fat—correlation with lipid profile and anthropometric measures.

**Table 5:** Diagnostic performance of various parameters in identifying high VF levels: AUC, sensitivity, specificity, and optimal cut-offs.

Test Parameter	AUC	95% Confidence Intervals		*p*-Value	Optimum Cut-Off	Sensitivity	Specificity	Younden’s Index
		Lower Bound	Upper Bound					
TGL	0.601	0.470	0.732	0.143	≥99.5	0.652	0.414	0.066
HDL Chol	0.650	0.525	0.775	0.030	≤45.65	0.655	0.478	0.123
WC	0.967	0.932	1	<0.0001	≥96.0	0.935	0.172	0.107
BMI	0.941	0.884	0.998	<0.0001	≥24.9	0.913	0.172	0.095
FEV1	0.533	0.400	0.666	0.636	≥50.5	0.543	0.414	−0.043
Criteria No	0.763	0.649	0.877	<0.0001	≥3.5	0.652	0.207	−0.141

**Table 6:** Spearman’s correlations between criteria number for MS and VF, TGL, HDL-Chol, FEV1, BMI, and WC.

Variable		VISCERAL FAT		TGL		HDL-Chol		BMI (kg/m^2^)		WC		Criteria Number for MS
Criteria number for MS	Spearman’s rho	0.539	***	0.552	***	−0.478	***	0.557	***	0.632	***	-
	*p*-value	<0.001		<0.001		<0.001		<0.001		<0.001		-

***—Statistically significant at *p* < 0.001.

## Data Availability

The original contributions presented in this study are included in the article; further inquiries can be directed to the corresponding author.

## Supplementary Materials

The following supporting information can be downloaded at: https://www.mdpi.com/article/10.3390/nu17061024/s1, Table S1: Diet components and human health impact. Refs. [56,57,58,59].
