# Supplementary material for: Visceral Fat, Metabolic Health, and Lifestyle Factors in Obstructive Bronchial Diseases: Insights from Bioelectrical Impedance Analysis
Source: Nutrients. 2025 Mar 14;17(6):1024. doi: 10.3390/nu17061024 (PMC11945945; doi:10.3390/nu17061024)
Supplement: Supplementary file 1 [file nutrients-17-01024-s001.zip › nutrients-3517811-supplementary.pdf]

Supplementary Materials

Table S1. Diet components and human health impact.

Diet Component	Human health impact	Food sources
**Carbohydrates**	Carbohydrates are the body's primary source of energy, playing an essential role in metabolism, brain function, and overall health. They are classified into simple (sugars) and complex (starches and fibers) carbohydrates, with the latter being more beneficial due to their slow digestion and sustained energy release. While essential for proper bodily functions, excessive intake of refined carbohydrates can contribute to obesity, diabetes, and cardiovascular diseases. Conversely, fiber-rich carbohydrates support gut health, regulate blood sugar levels, and reduce the risk of chronic diseases. A balanced intake of quality carbohydrates is key to maintaining optimal health.	Healthy sources: whole grains, fruits, vegetables, legumes, nuts, seeds, dairy products<br>Less healthy sources (to consume in moderation): refined grains (white bread, white rice, pasta made from refined flour), sugary foods (candies, pastries, sodas, desserts), processed snacks (chips, crackers, instant noodles)
**Proteins**	Proteins are essential macronutrients that support muscle growth, tissue repair, enzyme and hormone production, and overall body function. They provide amino acids, some of which are essential and must be obtained from the diet. Adequate protein intake is essential for immune function, metabolism, and maintaining lean body mass. While high-quality proteins contribute to good health, excessive intake of processed or red meats may increase the risk of chronic diseases. A balanced diet with diverse protein sources is key to optimal well-being.	Healthy sources: lean meats (chicken, turkey), fish (salmon, tuna, sardines), eggs, dairy (Greek yogurt, cottage cheese, milk), legumes (lentils, chickpeas, black beans), soy products (tofu, tempeh, edamame), quinoa, nuts (almonds, walnuts), seeds (chia, flax, pumpkin)<br>Less healthy sources (to consume in moderation): processed meats (sausages, hot dogs, bacon, deli meats), fried and fast-food proteins (deep-fried chicken, processed cheese, high-fat dairy).
**Fats**	Fats are essential macronutrients that provide energy, support cell function, and help absorb fat-soluble vitamins (A, D, E, K). They play a key role in brain health, hormone production, and reducing	Healthy sources (monounsaturated and polyunsaturated fats): olive oil,

	inflammation. However, the type of fat consumed matters—healthy fats promote heart health, while unhealthy fats increase the risk of cardiovascular diseases and obesity. A balanced diet should prioritize unsaturated fats while limiting saturated and trans fats.	avocados, almonds, cashews, peanuts, fatty fish (salmon, sardines, mackerel), flaxseeds, chia seeds, walnuts, sunflower seeds.<br>Less healthy sources (to consume in moderation): saturated fats (fatty cuts of red meat, butter, lard, palm oil), trans fats (processed snacks, fried foods, margarine, hydrogenated oils).
**Vitamins**	Vitamins are essential micronutrients that regulate various bodily functions, including metabolism, immunity, and cell growth. They play an important role in energy production, vision, bone health, and red blood cell formation. While a balanced diet provides most vitamins, deficiencies can lead to health problems such as weakened immunity, poor bone health, and fatigue. There are two types of vitamins: fat-soluble (A, D, E, K), which are stored in the body, and water-soluble (B-complex, C), which need regular replenishment.	whole grains, fruits, vegetables, legumes, nuts, seeds, dairy products, eggs, meat, seafood, fish
**Minerals**	Minerals are essential nutrients that support various bodily functions, including bone strength, nerve signaling, muscle contraction, and fluid balance. They are divided into macrominerals (needed in larger amounts) and trace minerals (required in smaller amounts but still vital). A well-balanced diet ensures proper mineral intake, while deficiencies can lead to health issues such as weakened bones, anemia, or impaired immune function.	whole grains, fruits, vegetables, legumes, nuts, seeds, dairy products, eggs, meat, seafood, fish
**Water**	Water is essential for life, making up about 60% of the human body. It plays a vital role in maintaining hydration, regulating body temperature, aiding digestion, transporting nutrients, and removing waste. Proper hydration supports brain function, joint lubrication, and cardiovascular health. Dehydration can lead to fatigue, headaches, kidney problems, and impaired cognitive function. While individual water needs vary, an average intake of 2-3 liters per day is generally recommended, depending on activity level and climate.	Water, fruits, vegetables, soups, beverages
**Fiber**	Dietary fiber is an essential component of a healthy diet, supporting digestion, gut health, and blood sugar regulation. It is classified into soluble fiber	Whole grains, legumes, fruits, vegetables, nuts, seeds

	(which dissolves in water and helps lower cholesterol and blood sugar levels) and insoluble fiber (which adds bulk to stool and promotes regular bowel movements). A high-fiber diet reduces the risk of constipation, heart disease, diabetes, and obesity while promoting a healthy gut microbiome. The recommended daily intake is around 25–30 grams for adults.	

References

1. Cena, H.; Calder, P.C. Defining a Healthy Diet: Evidence for The Role of Contemporary Dietary Patterns in Health and Disease. Nutrients. 2020, 12(2):334. doi: 10.3390/nu12020334.
2. Probst, Y.C.; Guan, V.X.; Kent, K. Dietary phytochemical intake from foods and health outcomes: A systematic review protocol and preliminary scoping. BMJ Open. 2017; 7:e013337. doi: 10.1136/bmjopen-2016-013337.
3. Ioniță-Mîndrican, C.-B.; Ziani, K.; Mititelu, M.; Oprea, E.; Neacșu, S.M.; Moroșan, E.; Dumitrescu, D.-E.; Roșca, A.C.; Drăgănescu, D.; Negrei, C. Therapeutic Benefits and Dietary Restrictions of Fiber Intake: A State of the Art Review. Nutrients, 2022, 14, 2641; https://doi.org/10.3390/nu14132641
4. Gorski, M.T.; Roberto, C.A. Public health policies to encourage healthy eating habits: recent perspectives. J Healthc Leadersh. 2015; 7:81-90. doi: 10.2147/JHL.S69188.
